# Intrinsic factors influencing help-seeking behaviour in an acute stroke situation

**DOI:** 10.1007/s13760-015-0555-4

**Published:** 2016-01-05

**Authors:** Elles Zock, Henk Kerkhoff, Ruud Peter Kleyweg, Diederik van de Beek

**Affiliations:** 1 Department of Neurology, Albert Schweitzer Hospital, Albert Schweitzerplaats 25, 3318 AT Dordrecht, The Netherlands; 2 Department of Neurology, Academic Medical Center, Amsterdam, The Netherlands

**Keywords:** Stroke, Acute treatment, Delay, Help-seeking behaviour, Knowledge, Qualitative research

## Abstract

The proportion of stroke patients eligible for intravenous or intra-arterial treatment is still limited because many patients do not seek medical help immediately after stroke onset. The aim of our study was to explore which intrinsic factors and considerations influence help-seeking behaviour of relatively healthy participants, confronted with stroke situations. Semi-structured interviews were conducted with 25 non-stroke participants aged 50 years or older. We presented 5 clinical stroke situations as if experienced by the participants themselves. Recognition and interpretation of symptoms were evaluated and various factors influencing help-seeking behaviour were explored in-depth. We used the thematic synthesis method for data analysis. Five themes influencing help-seeking behaviour in a stroke situation were identified: influence of knowledge, views about seriousness, ideas about illness and health, attitudes towards others and beliefs about the emergency medical system. A correct recognition of stroke symptoms or a correct interpretation of the stroke situations did not automatically result in seeking medical help. Interestingly, similar factors could lead to different types of actions between participants. Many intrinsic, as well as social and environmental factors are of influence on help-seeking behaviour in an acute stroke situation. All these factors seem to play a complex role in help-seeking behaviour with considerable inter-individual variations. Accomplishing more patients eligible for acute stroke treatment, future research should focus on better understanding of all factors at various levels grounded in a theory of help-seeking behaviour.

**Electronic supplementary material:**

The online version of this article (doi:10.1007/s13760-015-0555-4) contains supplementary material, which is available to authorized users.

## Introduction

Intravenous or intra-arterial treatment (thrombolysis, mechanical treatment or both) is effective for patients with acute ischemic stroke with a time window after stroke onset not exceeding 4.5 or 6 h, respectively. [1–5]. The proportion of patients eligible for these therapies is still limited because many patients do not seek medical help immediately after stroke onset [6–9]. Stroke educational campaigns have been launched to enhance stroke knowledge and awareness, focusing on the importance of early treatment [8, 10–12]. These campaigns have had temporary or limited effect on the proportion of patients eligible for alteplase [13, 14]. Apparently, knowledge about stroke warning signs does not automatically result in help-seeking behaviour [15, 16].

In the past two decades many studies focused on the role of factors other than knowledge in help-seeking behaviour after stroke. For example culture, age, socioeconomic status, educational level and stroke severity were identified [17–21]. Other studies focused on more intrinsic factors, such as personal beliefs, values, and attitudes [12, 15, 22–25]. These intrinsic factors seem of great importance in the process of help-seeking behaviour after stroke onset. However, these studies varied in design, results, theoretical grounding and, moreover, patients with recent stroke were studied. Recall bias could have been present. We believe we first must return to the process of decision-making in relatively healthy persons. This will provide one step in subsequently understanding the complex process of help-seeking behaviour in a stroke situation and may help in a more systematic, theoretical design of successful stroke campaigns [26].

In this qualitative study, we explored which intrinsic factors and considerations influence help-seeking behaviour of relatively healthy participants, confronted with stroke situations.

## Methods

### Design and population

We used a qualitative study design involving individual semi-structured and in-depth interviews. The ethics committee of the Albert Schweitzer Hospital approved the study protocol. Participants were patients from the outpatient orthopaedic department of the Albert Schweitzer Hospital Dordrecht, the Netherlands, between September 2013 and October 2013. We included participants aged 50 years or more with equal representation of men and women. Subjects were explained that the study aimed to explore how they deal with health-related problems. The interviewer was blinded for the medical history of the participants. People with a language barrier were excluded from the interviews. Interviews were held in a quiet separate room and audio-recorded. All subjects gave written informed consent. The interviewer (EZ) was trained for this type of research and interview methods. She had a 5-year clinical experience with stroke patients. The interviews lasted about 30 min.

### Interview

During the interviews an interview guide was used. Each interview started with obtaining demographic data, as age, gender, ethnicity, working status and educational level. Data about a history of stroke or strokes among relatives or friends were obtained. Help-seeking behaviour in stroke-like situations was explored with semi-structured and in-depth interviews. We presented five real-stroke situations with a variation in stroke symptoms, severity and social setting, in a random sequence (see appendix). We used original patient descriptions, derived from stroke patients in our emergency medical unit. We asked the participants to imagine that these situations were happening to themselves. We defined recognition as noticing something was wrong the body or impaired functioning [25]. Interpretation was defined as interpreting the noticed symptoms in terms of a disease [25]. Participants were asked the following questions; more answers were accepted: (1) what they thought was happening in that situation, (2) whether the symptoms were associated with a specific disease, (3) whether they thought there was a treatment and if so, what kind of treatment. All participants were asked which symptoms and risk factors are generally associated with a stroke. In-depth interviewing was used to explore the action that the participant would take and the reasons and factors leading to such an action.

### Data analysis

Descriptive statistics were used for analysing demographic and quantitative data. All interviews were transcribed verbatim and entered in NVivo (version 10, QSR International), a software program for qualitative data analysis [27]. We used the thematic synthesis method: coding text line-by-line, development of descriptive themes and generation of analytical themes [28, 29]. Three investigators (EZ, HK, RK) read the transcriptions. First each interview was coded by the head coder (EZ) and alternating by the two co-coders (HK, RK) independently from the head coder. Secondly, coded interviews were discussed plenary using the constant comparison method. When the two coders disagreed, the third coder made a decision about the final coding of the text. Sample size was determined by the principal of theoretical saturation [30]. The criteria for rigour in qualitative research as described by Shenton et al. were adhered [31].

## Results

Twenty-five persons were interviewed before reaching theoretical saturation. Mean age was 68 years (range 54–78), 12 were men and all were Caucasian (Table 1). The majority of the participants completed intermediate vocational education. One participant had a history of ischemic stroke and 17 knew someone with a stroke. Five major themes in help-seeking behaviour in stroke situations were identified: influence of knowledge, views about seriousness, attitudes towards others, ideas about illness and health and beliefs about the emergency medical system. We identified four types of action: asking for medical help, asking for non-medical help, wait and see or a chain reaction. Asking for medical help consisted of consulting the general practitioner (GP) or the emergency medical services. Asking or calling for non-medical help often resulted in consulting a bystander or contacting with family. A chain reaction consisted of different sequential actions as for example going home, calling family and eventually consulting the GP.

**Table 1:** Participant characteristics (*N* = 25)

Men (*N*)	12
Mean age (years)	68.7
Ethnicity (*N*)	
Caucasian	25
Education (*N*)	
Primary school	5
Lower vocational education	6
Intermediate vocational education	10
Higher vocational education	1
University education	3
Prior stroke (*N*)	1
Stroke in relatives, friends (*N*)	
None	8
Parent (s)	5
Brother/sister (s)	3
Child(ren)	1
Other family member (s)	4
Friend/neighbour (s)	4

## Influence of knowledge

### Recognition and interpretation

Five stroke situations were presented to each of the 25 participants, which yielded a total of 125 stroke cases. A stroke was directly interpreted as the cause in 32 cases. Furthermore, in an additional 24 cases a stroke was mentioned as one of the possible causes. Two participants never thought of a stroke or description of a circulation problem in the brain in the 5 presented situations.

Many participants recognized symptoms as a problem located in the brain, but misinterpreted the exact cause or disease categorizing it as dementia, vertigo, a non-specific circulation problem or a brain tumour (Table 2). Some interpreted it as a ‘blockade (005)’ or a ‘trembling hard disk (019)’ [quotes per theme and patient identification number (PID) are presented in Table 3 (supplementary)]. In many situations, participants categorized the problem as musculoskeletal, nerve compression, or pulmonary embolism. Sometimes blood regulation disturbances or a heart attack were presumed. Some recognized a left-sided facial droop and arm paresis correctly as warning signs, but interpreted this as a heart problem because of the belief that all heart problems result in a problem of the arm (011). In nineteen cases, the participant had no idea what was going on.

**Table 2:** Interpretations of presented symptoms (N)*

Cerebral problem	
TIA/ Stroke	66
Problem in head or brain	17
Brain tumour	6
Dementia	6
Problem of the eyes	2
Migraine	1
Circulation problem	
Heart attack	22
Blood pressure problem	13
Standing up to fast	2
Embolism of the heart	2
Oxygen deficiency	2
No idea	27
Musculoskeletal problem	
Muscular	3
Degenerative	2
Overload	2
Fracture	1
Cancer	1
General description	
Something wrong	6
Failure of nature	2
Nerve compression	
In the lumbar spine	5
In the leg	1
Consciousness problem	5
Respiration problem	
Oxygen deficiency	2
Pulmonary embolism	1
Other	
Ear, nose, throat problem	2
Glucose problem	2
Balance problem	2
Medication problem	1
Notion problem of other person	1
Allergy	1

*More answers were accepted

A correct recognition of symptoms or interpretation of a stroke situation did not automatically lead to immediate action (003). In 15 of the 32 times the participant straightforward interpreted the situation as a stroke; however, calling the emergency medical number was not considered. Some participants only recognized that something was wrong but waited to call for any help. Personal experiences of participants with earlier spontaneous disappearance of symptoms resulted in a wait and see strategy. Ten participants never suggested calling the emergency medical number. Misinterpretation or having no idea of the presented situation could lead to immediate action (007).

Two participants were aware of a time window for stroke treatment and the need for immediate medical help. These participants interpreted the majority of situations correctly as a stroke.

### Handling knowledge

Many participants had no interest in gaining knowledge about stroke and treatment options. They explained they might be interested in such information when they had a stroke themselves (023). Others felt that information about stroke warning signs should be left to the physicians. Only one participant was aware of a stroke campaign. He felt obligated knowing symptoms in case someone around him needed his help (021). He did not interpret all the stroke situations correctly. Other participants had gained stroke knowledge from weekly magazines, but mostly from relatives or friends with stroke. The latter group recognized stroke-warning signs, but only if presented signs matched with their experience (003). Most participants could name one or more risk factors for stroke, most commonly overweight, smoking, high blood pressure and alcohol abuse. Some participants could not specify risk factors, but presumed that general unhealthy living, too much work, not enough sleep and stress would increase the risk for stroke.

## Views about seriousness

Participants differed in their view of seriousness, irrespective of recognition of stroke. In general, symptom evolvement was seen as important. Symptoms that would disappear over minutes were judged as less serious than symptoms lasting for hours or days (025). None of the participants with a wait-and-see strategy imagined possible consequences. In general, participants with knowledge about possible consequences would call the emergency medical number. One participant explained that development of serious symptoms would prompt him to call a doctor, but was not able to describe the nature of serious symptoms. Some participants related seriousness to functionality in daily life. There were contrasting attitudes about speech problems. For some participants this implied as serious while for others it would not impact their daily activities.

Judgment of seriousness and the cause of symptoms was influenced by the medical history of participants. Vertigo, coordination problems and diplopia were not considered to be a TIA, stroke or other serious situation because many participants had a history of benign vertigo (010). One participant with known lumbar nerve compression was instructed to call an ambulance in case of paresis of his leg. He recognized this situation in one of our stroke cases. Participants with arthrosis had suffered from leg dysfunction and were not alarmed in this specific situation (009). In general, when symptoms were judged as serious, medical help was sought; however, views about seriousness differed widely between participants.

## Attitudes towards others

Many participants needed advice and asked for non-medical help from someone else (002). Having someone around in case of worsening was also another important motivation for asking non-medical help. For many it was important to be at home for further consideration and subsequent action (009). Several participants with a wait-and-see strategy indicated that they did not want to be a burden for someone else. Despite correct interpretation of the stroke situation they would try to resolve the problem by themselves first (018).

## Ideas about illness and health

The majority of participants noticed that they were raised not to complain so easily (012). Many were convinced that symptoms would resolve spontaneously, ‘what comes will go’ and, therefore, no action would be undertaken (025). Others would not link symptoms or complaints with disease because they were healthy. Some believed that they were not ill, as long as someone else had not told them (009). This resulted in an ignoring strategy and, therefore, a wait and see policy. They would eventually deal with the problem, until it was too prominent to ignore (023). Some participants had clear ideas when to call for medical help: pain, a fracture, shortness of breath (024), chest pain or red colour of the skin. Long duration of symptoms, recurrence of symptoms or impaired functioning were reasons for seeking medical attention. Another reason to call for a doctor was reassurance for partner or family (014). Some had experienced a severe disease in the past, which changed their attitude towards illness. Since then, they sought medical attention in case of any symptoms (022).

## Beliefs about the medical emergency system

Different opinions existed about the accessibility of the emergency medical number. Most of the participants would consult their GP first, even when the situation was judged as being serious or as having a stroke (018). One participant thought that medical costs were not insured if the GP was not consulted first. Another important reason to call or to consult the GP was clarification of a medical problem or referral towards a medical specialist. Participants, who found it difficult to explain the exact problem in the presented situations, hesitated whether it was appropriate to call the emergency medical number or GP (008). Some participants believed that physicians in general would not appreciate their ideas or knowledge about diseases (019). For this reason they found it not important for themselves to have knowledge about diseases.

## Discussion

Our study showed that although many participants interpreted our stroke situations correctly, this did not prompt immediately help-seeking behaviour. Previous research showed that the decision of patients to seek immediate medical help results from a complex interaction of several factors. Behaviour in a stroke situation depended on how symptoms were perceived, prior experience of illness, cultural norms and values within the community [23]. Our results support these findings but we explored also additional important factors and considerations, such as not wanting to bother somebody, hesitation to make a decision, having learned not to complain so easily or a tendency not to take action until symptoms are too prominent to ignore. Some factors are intrinsic, where others are more socially or environmentally driven. This shows the importance of understanding how all these factors at multiple levels result in help seeking behaviour [26].

Remarkable statements were made about not being perceptive for information about stroke. Although knowledge about stroke warning signs alone does not automatically result in immediate action, the importance of sufficient knowledge must not be underestimated. One of the challenges is how to optimize knowledge about stroke signs if people are not interested in it. Understanding the phenomenon of being non-perceptive and focusing on how to reach those people may be crucial for development of successful stroke campaigns in the future.

We made a distinction between recognition and interpretation of stroke symptoms. This is in contrast with many previous studies that used recognition of stroke warning signs directly as the interpretation of these signs as a stroke and made no difference between recognition and interpretation [32–34]. We used the definitions as reported by Moloczij et al. [25]. Some people indicated to seek help after recognition, independent of an adequate interpretation. For others, thinking about unknown consequences led to an adequate action. The distinction between recognition and interpretation is important because it underlines that decision-making is far from a linear process. Moloczij et al. introduced a model of help seeking behaviour after stroke onset using interrelated steps: starting with recognition, interpretation, negotiation and finally action [25]. Our data suggest that even this complex model is oversimplified. It seems that many factors are present at the same time and have an interaction. Therefore, a sequential decision model could not be arranged.

An important drawback of most studies about stroke knowledge is that questionnaires in medical terms to explore recognition were used [35–37]. This, however, is a recall task of stroke knowledge rather than an exploration of help seeking behaviour in stroke. When people experience a stroke by themselves or as a bystander, they see someone falling down, hear someone speaking with strange words, or hear somebody complaining about visual problems. In contrast, our qualitative research design with presentation of stroke situations in lay terms enabled us to explore the dimensions of recognition and interpretation in a more realistic way.

We classified calling the GP or calling the emergency medical number as one type of action, asking for medical help. Although this is an adequate action in comparison with for example a wait and see strategy, we realize calling the GP instead of the emergency medical number, results in a delay. Therefore, the message should be to call the emergency medical number immediately after stroke onset.

A limitation of our study is that it was conducted in a hospital setting. We intended to include relatively healthy participants facing a stroke situation as occurring in real life. Ideally, the participants would have been recruited in public places. For logistic reasons and trustworthiness of our data, we choose for a hospital setting. Although participants had no information about the subject of the interviews, it is possible that the hospital setting influenced their opinions and answers. We choose for orthopaedic patients, because most of their problems have not the seriousness to call for immediate medical help. An important drawback of our study is that in real life the decision process may be influenced not only by symptoms patients actually experience, but also by emotional and cognitive impairment, secondary to a stroke and reaction of possible bystanders. All our participants were Caucasian. Differences related to ethnicity in help-seeking behaviour have been studied with inconsistent results [18, 38]. Perception of health and illness, beliefs about the health medical system and attitude towards others, however, may differ between ethnicities. Further study is needed to explore such ethnicity-related diversities.

In conclusion, many intrinsic as well as social and environmental factors influence help-seeking behaviour after stroke onset. Decision-making after stroke onset is not a linear process with considerable inter-individual variations. It is important to realize that factors concerning views about the seriousness of the situation, attitude towards others, beliefs about illness and health, beliefs about the medical emergency system are of similar or even more importance than a correct interpretation of stroke symptoms themselves. A shift in the message of future stroke campaigns seems essential in accomplishing more patients eligible for acute stroke treatment. Future research in real-stroke situations should focus on better understanding of all factors at various levels grounded in a theory of help-seeking behaviour.

## Electronic supplementary material

Below is the link to the electronic supplementary material.

Supplementary material 1 (docx 83 kb)

Supplementary material 2 (docx 92 kb)
