# Supplementary material for: Intrinsic factors influencing help-seeking behaviour in an acute stroke situation
Source: Acta Neurol Belg. 2016 Jan 5;116:295–301. doi: 10.1007/s13760-015-0555-4 (PMC4989004; doi:10.1007/s13760-015-0555-4)
Supplement: Supplementary file 1 — Supplementary material 1 (docx 83 kb) [file 13760_2015_555_MOESM1_ESM.docx]

**APPENDIX 1**. Stroke like situations

Case 1 While walking outside with your dog, you suddenly become dizzy. It feels as if the world is turning around you. You are unable to stand on your own and must grasp a tree for support. When you continue walking it is as if being drunk. Looking at your dog you see her twice. You sit down but the symptoms do not disappear.

Case 2 You just came out of bed and took a shower. Suddenly you bend through one leg. You cannot hold the showerhead in your hand and it falls down. You just manage to keep standing on your feet and come out of the shower with difficulty.

Case 3 You are in the gym doing your exercises. When you walk to the next exercise, you suddenly get a very severe, cutting like, headache and become nauseous. You are able to sit down quickly. By setting the weight for the next apparatus, you are not able to move your right hand properly. You have lost the coordination of that arm.

Case 4 At home you are preparing a meal for your family. During cooking you suddenly notice that the right side of your tongue and cheek feel numb. It also seems some saliva is dropping out of your mouth. Arriving at the table, your son tells you that your mouth is standing in a different way, as if the right side has dropped. You try to speak, but you are troubled. It is like your tongue is swollen. Your son cannot understand you because of a slurred speech.

Case 5 You are in a shop buying cloths. You are holding two garments with different sizes and need a shop assistant. When asking her the meaning of the two different sizes, you cannot speak normally. Finishing a sentence is not possible. The only thing you can say is the size. The shop assistant does not understand you and asks what you mean. Again you try to ask your question, but you cannot speak.
