# Supplementary material for: Intrinsic factors influencing help-seeking behaviour in an acute stroke situation
Source: Acta Neurol Belg. 2016 Jan 5;116:295–301. doi: 10.1007/s13760-015-0555-4 (PMC4989004; doi:10.1007/s13760-015-0555-4)
Supplement: Supplementary file 2 — Supplementary material 2 (docx 92 kb) [file 13760_2015_555_MOESM2_ESM.docx]

| Table 3. Quotes per theme | | |
| --- | --- | --- |
| **Theme** | **PID** | **Quote** (transcribed verbatim) |
| Influence of knowledge |  |  |
| Recognition, interpretation | 005  019  011  003  007 | EZ: What do you think is going on at that moment? PT: It might be something in the brain, when you have unexpectedly failure of knee function and having no complaints. It seems to me that something has a blockade, uhm, for a moment.  PT: So somewhere here (pointing at head) a screw might be unattached (laughing). EZ: A screw unattached, yes? PT: A trembling hard disk for a moment.  PT: Because of that …, because if that arm, yes, I… (EZ: Yes). My father was a cardiac patient and he always had a problem of his arm so (EZ: Yes), that is why I think this.  PT: Yes, but I don’t believe I immediately would undertake some action. EZ: Yes. PT: And then, somewhere in the back of your head, it goes through your mind that you think; I suppose I have had a stroke.  PT: And if someone else is telling me that I’m speaking strangely, than I would suggest consulting the doctor. I would ask if he could… if we could stop by. |
| Handling knowledge | 023  021  003 | PT: Sometimes you read or see something, but it goes in here and out there (pointing at ears). EZ: What is the reason for that? PT: Well, maybe because I, uhm, have no need for it at that moment. And when you are feeling miserable at a certain moment and you notice that what is going on in the head does not resolve, maybe you are more interested at that moment than when you have no complaints.  PT: I don’t want to be helpless when something like that happens to my wife or children. That I feel like; ‘Help, I wished I had’ and that I have no idea what to do in that situation. I don’t want to blame myself for that.  EZ: Do you know what the stroke campaign was about? What a stroke means? PT: Yes, that it is a problem coming out of the brain. Uhm, shaking, walking problems, empty talking and strange watching, although that is what I remember from my father a long time ago. |
| Views about seriousness | 025  010  009 | PT: But when the symptoms disappear, there is no problem, but when they stay (EZ: Yes?), then you have to make a call.  PT: Because I had it once while I was walking. I overestimated walking and really had exhausted my body.  PT: Yes, the bones, right? EZ: Hm. PT: It all worsens, so maybe it is the arthrosis that makes you fall. I have had that more often, maybe it has something to do with this. |
| Attitudes towards others | 002  009  018 | PT: I think calling for help from, uhm, someone who is supervising. EZ: Yes, what would you want that person to do? (…) PT: Yes, uhm, I think he will ask what is going on and I will say my arm is not functioning (EZ: hm) and I hope he or she knows something, call for …, or can tell me what to do.  PT: You better can go home, you are safe there, I believe. EZ: Yes, is that what is important for you? PT: Yes, I think it is, home is the place where you are safe and you can call your husband or someone else, when you think you recover or the situation worsens.  PT: I would search for the telephone, call my children and think that it is going to be all right. EZ: Why do you think that? PT: Because I have always been self dependent and I feel troubled things to be left to others. |
| Ideas about illness and health | 012  025  009  023  024  014  022 | EZ: And what is the reason that you don’t call for help so easily? PT: Yes, I believe that symptoms will recover (EZ: Yes), so, uhm, that’s what you have been taught, not complaining, just deal with it.  PT: You assume that it came by itself so it will disappear by itself.  PT: I say listen, I am not going to make myself crazy as long as I don’t know what is going on (EZ: No). I say, what does that bring me? It is not there and as long as it is not there, it is not present.  PT: When it happens several times every week (EZ: Yes), then I will think something is not right and I would visit the GP.  EZ: Which signals ask for immediate action in your opinion? PT: When I am in danger to choke. So when I have problems with my breathing, I cannot control it myself (EZ: Yes, yes), then I will call for a doctor.  EZ: And on a certain point you will consult a doctor. Is this because of pain, or impairment, or? PT: No, I will do it for her (pointing at his wife)  PT: I believe I am more careful now. Faster I think, faster consulting a doctor instead of taking an aspirin. |
| Beliefs about the medical emergency system | 018  008  019 | EZ: In your opinion, what has to be done? PT: In this country you must, uhm, call the GP first.  PT: I would not call so fast 123, or 112. EZ: No, why not in this particular situation? PT: Yes, because I don’t know what is happening to me.  EZ: Does your level of knowledge about health problems make difference in action taking? PT: That is tricky, because, uhm, you are the doctor. I consult you and say: ‘doctor, I think I have this or that’. What will the doctor think of me? There we have again a “google know-it-all”. No, the doctor does not like you know such things. |
| PID = patient identification number, EZ = interviewer, PT = participant, GP = general practitioner | | |
